# Diagnosis of Single- or Multiple-Canal Benign Paroxysmal Positional Vertigo according to the Type of Nystagmus

**DOI:** 10.1155/2011/483965

**Published:** 2011-07-14

**Authors:** Dimitris G. Balatsouras, George Koukoutsis, Panayotis Ganelis, George S. Korres, Antonis Kaberos

**Affiliations:** ^1^ENT Department, Tzanio General Hospital of Piraeus, Afentouli 1 and Zanni, 18536 Piraeus, Greece; ^2^ENT Department, University General Hospital Attikon, 1 Rimini Street, Haidari, 12462 Athens, Greece

## Abstract

Benign paroxysmal positional vertigo (BPPV) is a common peripheral vestibular disorder encountered in primary care and specialist otolaryngology and neurology clinics. It is associated with a characteristic paroxysmal positional nystagmus, which can be elicited with specific diagnostic positional maneuvers, such as the Dix-Hallpike test and the supine roll test. Current clinical research focused on diagnosing and treating various types of BPPV, according to the semicircular canal involved and according to the implicated pathogenetic mechanism. Cases of multiple-canal BPPV have been specifically investigated because until recently these were resistant to treatment with standard canalith repositioning procedures. Probably, the most significant factor in diagnosis of the type of BPPV is observation of the provoked nystagmus, during the diagnostic positional maneuvers. We describe in detail the various types of nystagmus, according to the canals involved, which are the keypoint to accurate diagnosis.

## 1. Introduction

Of all the inner ear disorders that can cause dizziness or vertigo, benign paroxysmal positional vertigo (BPPV) is by far the most common [1]. Additionally, it is a condition that in most instances may be easily diagnosed and treated, with a simple office-based procedure [2]. Since the initial description by Bárány in 1921 [3], there have been major advances in the understanding of this common condition. Recently, modern clinical research focused on diagnosing and treating various types of BPPV, according to the semicircular canal involved and according to the implicated pathogenetic mechanism. Multiple-canal BPPV has been specifically investigated, as the main source of various atypical forms of the disease, which until now were resistant to treatment with standard canalith repositioning procedures (CRPs) [4, 5]. The purpose of this paper is to present the data regarding the various types of nystagmus produced during the diagnostic maneuvers of BPPV, which in conjunction with the patient's history and symptoms, will help in obtaining accurate diagnosis and appropriate treatment. In all subsequent discussions, the various types of nystagmus will be described according to their fast phase, relative to the patient's perspective (e.g., as horizontal nystagmus with a fast phase beating towards the patient's right ear is termed rightward horizontal nystagmus and a rightward torsional nystagmus, which is beating towards the patient's right ear, is a counterclockwise nystagmus, as seen by the observer).

## 2. Unilateral Posterior Canal BPPV

This is the most common type of BPPV, accounting for up to 90% of the patients [6]. The Dix-Hallpike provoking maneuver is used to diagnose the disease by moving the patient rapidly from a sitting position to a position of head hanging with each ear alternately undermost. Posterior semicircular canal involvement is proved from the type of the visually observed paroxysmal positioning nystagmus, which is beating towards the undermost and affected ear, with a torsional component clockwise when following leftward movement, or counterclockwise, when following rightward movement [7]. Typically an upbeating nystagmus component is superimposed, resulting in a mixed torsional-vertical eye movement. Intense vertigo in conjunction with this pattern of nystagmus and the additional characteristics of a short latency, limited duration, intensity characterized by crescendo and decrescendo element, reversal on returning to the upright position, and fatiguability on repetitive provocation may easily establish the diagnosis of posterior canal BPPV. 

Canalolithiasis is the implicated pathogenetic mechanism for this disorder, characterized by the presence of free floating debris within the posterior semicircular canal, detached from the otoconial layer by degeneration or head trauma [8]. The otoconia gravitates into the posterior canal, where it forms a plug floating in its nonampullary branch. In the provoking Dix-Hallpike position the endolymph pulls on the cupula, because the free-floating otoconia falls under the influence of gravity. In the vertical canals, ampullofugal deflection produces an excitatory response. This would cause an abrupt onset of vertigo and the typical nystagmus described previously. Nystagmus latency is explained by inertia of the clot. The cupula deflection ends when the clot reaches its lowest position and accounts for the limited duration of the nystagmus. Fatigue is due to dispersion of the clot particles and reactivation after bedrest is caused by renewed clot formation. 

An alternative pathogenetic theory, the cupulolithiasis of the posterior canal, may account for a small rate of cases with posterior canal BPPV [7, 8]. According to this, otoconia with a specific gravity greater than endolymph from a degenerating utricular macula settle on the cupula of the posterior canal, rendering it sensitive to gravity. Certain head movements may then produce inappropriate endolymph-cupula displacement, causing nystagmus and vertigo, which in this case is of longer duration. The latency before the onset of nystagmus reflects the inertia of the otoconial mass and the cupula, and the fatiguability is presumably due to dispersal of the debris attached to the cupula or even to central vestibular adaptation. 

**Figure 1:**
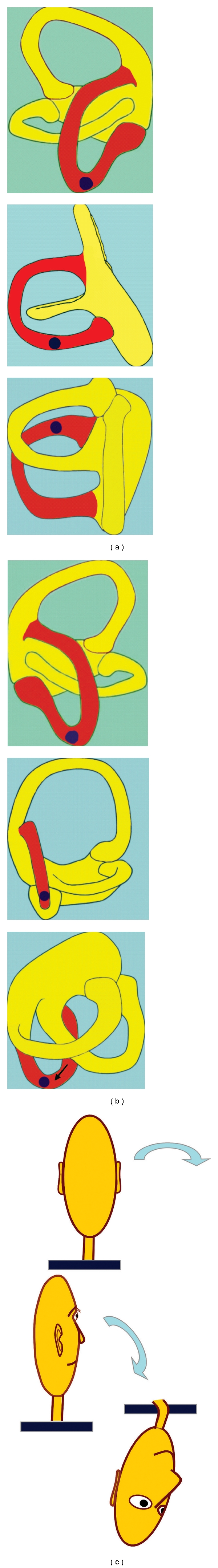
Stimulation of the posterior semicircular canal during the right Dix-Hallpike maneuver (c). On (a,b), the semicircular canals of the left and right ears, respectively, are shown. When otoconia is present in the ipsilateral (right) posterior canal (b), the maneuver causes its movement along the lumen of the canal, inducing BPPV. When otoconia is present in the contralateral (left) posterior canal (a), the maneuver does not cause any movement of the otoconia, because the head rotates orthogonally to the plane of the involved canal and the maneuver is negative.

The previously described profile of nystagmus correlates with the known neuromuscular pathways that arise from stimulation of the posterior canal ampullary nerves in animal models and humans [9, 10]. It should be noticed that the character of nystagmus changes with the direction of gaze, which is explained by contraction of the ipsilateral superior oblique and contralateral inferior rectus, following the stimulation of the posterior canal. When the patient lies in the lateral head hanging position, if he looks towards the uppermost unaffected ear, the axes of these two extraocular muscles nearly coincide, resulting in movement of the eyes in a vertical plane with predominance of the vertical component of the nystagmus. When looking towards the lowermost involved ear, the axes of these two muscles are nearly at right angles with the direction of the gaze, and their contraction results in apogeotropic rolling of the upper pole of the eye (slow phase) and predominance of the torsional component of the nystagmus with geotropic fast phase [11]. The Dix-Hallpike maneuver is usually positive only when performed with the involved ear undermost and negative on the contralateral side, permitting thus easy localization of the side of the lesion (Figure 1). It should be also noticed, that posterior canal paroxysmal positional nystagmus is dissociated, with the torsional component being more evident in the ipsilateral eye, and the vertical upbeating component more evident in the contralateral eye, which can be explained by different angle of insertion of the oblique and rectus muscles [12, 13]. In Table 1, the various types of BPPV nystagmus are described, according to the involved semicircular canal and the side of involvement.

## 3. Unilateral Horizontal Canal BPPV

BPPV originating from stimulation of the horizontal semicircular canal is the second most common type of BPPV, accounting for approximately 5–15% of the patients [6, 14–16] but its frequency has been occasionally reported up to 30% [17]. The patient can get up or lie down, bend or straighten up with minimal complaints, but turning the head to either side in the supine position provokes intense vertigo, and a purely horizontal paroxysmal positioning nystagmus. Vertigo may be more intense than in posterior canal involvement and is usually associated with severe autonomic symptoms. Two major types of horizontal canal BPPV may be distinguished, according to the pathogenetic mechanism of canalolithiasis or cupulolithiasis. Canalolithiasis may manifest as BPPV with geotropic paroxysmal nystagmus, and less frequently with apogeotropic nystagmus, when the otoliths are located in the short arm of the horizontal semicircular canal, near the ampulla. Cupulolithiasis manifests as apogeotropic persistent nystagmus, commonly with absence of latency during the supine roll test.

### 3.1. Geotropic Horizontal Canal BPPV

**Figure 2:**
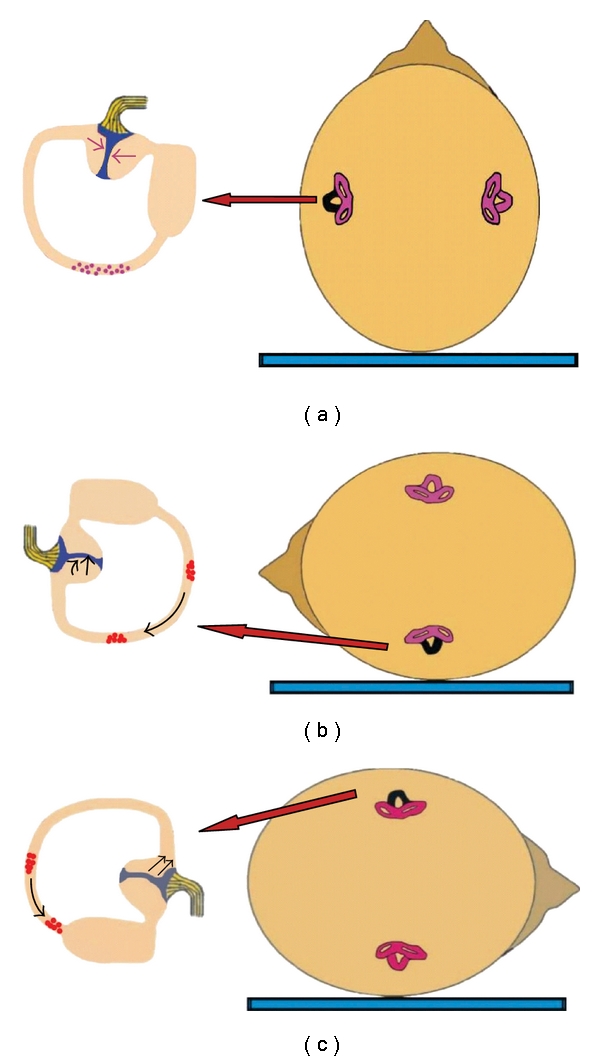
Mechanism of canalolithiasis of BPPV of the horizontal semicircular canal, when the left ear is affected (the involved left horizontal canal is colored black). (a) Patient in supine position with debris in the posterior part of the left horizontal canal. (b) When rotating the head towards the affected side, particles move towards the ampulla, producing an ampullopetal flow and triggering intense geotropic horizontal nystagmus. (c) When rotating the head towards the healthy side, particles fall in the opposite direction, causing an ampullofugal flow and triggering nystagmus beating again towards the ground, but less intense.

This is the most common type of horizontal canal involvement, accounting for approximately 2/3 of the cases [2, 18]. The canalolithiasis theory can also explain this BPPV variant. Degenerative debris enter the nonampullary side of the pathological horizontal canal when the patient lies supine (Figure 2(a)). Diagnosis is made by the supine roll test, turning the head from the supine to either lateral position. When rotating the head to the pathological side (Figure 2(b)), gravity and the angular head acceleration make the mass descend in the canal towards the ampulla. The movement of the clot continues until the deepest position is reached and provokes an ampullopetal deviation of the cupula, resulting in a burst of nystagmus towards the ground. When maintaining the head rotation to the pathological side, a burst of nystagmus with opposite fast phase (away from the ground) can be seen. This may be attributed to short-term adaptation of the vestibule-ocular reflex [19] or to an inversion of the direction of clot movement, due to a spontaneous reflux of endolymph between debris in the canal and membranous walls, facilitated by the elastic forces of the cupula. Another possibility is mixed canalolithiasis-cupulolithiasis, which may initially manifest as intense paroxysmal geotropical nystagmus owed to canalolithiasis, superimposed over the opposite nystagmus of cupulolithiasis, followed by the apogeotropical persistent nystagmus of cupulolithiasis. The same type of nystagmus can also be obtained by returning the head to the original position. When the head is rotated to the healthy side (Figure 2(c)), the mass is displaced further towards the nonampullary end of the canal with an ampullofugal displacement of the cupula, resulting in a nystagmus of lower intensity, beating towards the ground. Latency is usually shorter in horizontal canal BPPV. To summarize, horizontal canal BPPV owed to canalolithiasis manifests as bilateral geotropic horizontal nystagmus, which is more pronounced in the pathological side. This type of nystagmus is characterized by a short latency, a very sudden onset, and a longer duration as compared with the paroxysmal nystagmus of the posterior canal.

### 3.2. Apogeotropic Horizontal Canal BPPV

**Figure 3:**
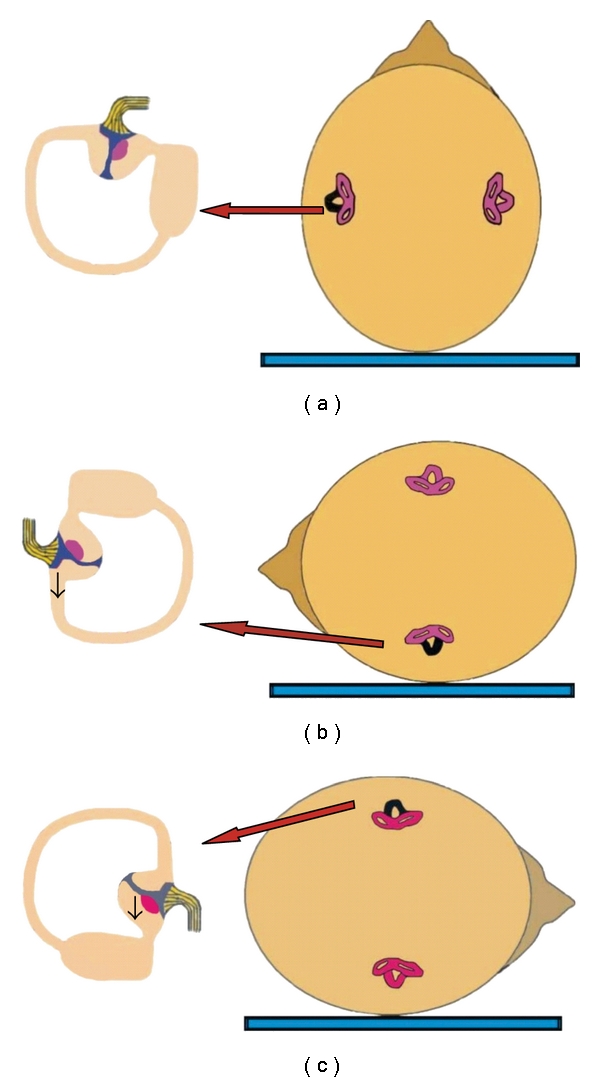
Mechanism of cupulolithiasis of BPPV of the horizontal semicircular canal, when the left ear is affected (the involved left horizontal canal is colored black). (a) Patient in supine position with debris adherent to the cupula of the left horizontal canal. (b) When rotating the head towards the affected side, the cupula will undergo an ampullofugal (inhibitory) deflection, triggering an apogeotropic horizontal nystagmus. (c) When rotating the head towards the healthy side, the cupula will undergo in ampullopetal (stimulatory) deflection, triggering a more intense apogeotropic nystagmus.

**Figure 4:**
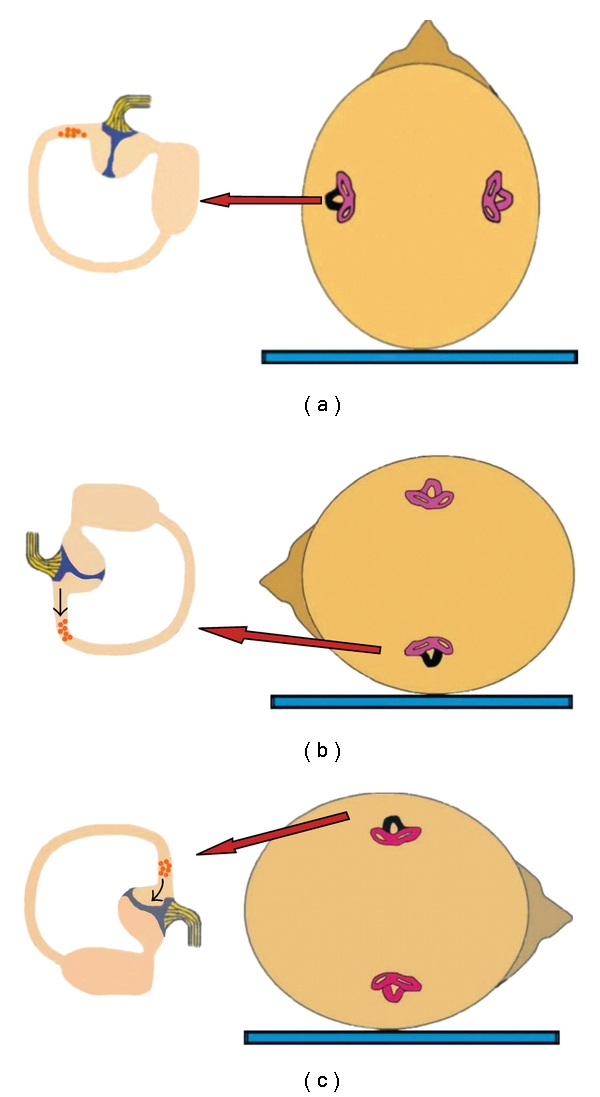
When canalolithiasis of the short arm of the horizontal semicircular canal near the ampulla occurs (left ear), a similar nystagmus (bilateral apogeotropic) as in the cupulolithiasis cases is observed during the supine roll test (the involved left horizontal canal is colored black). (a) Patient in supine position with debris in the short arm of the left horizontal canal. (b) When rotating the head towards the affected side, the particles move away from the cupula, which will undergo an ampullofugal (inhibitory) deflection, triggering an apogeotropic horizontal nystagmus. (c) When rotating the head towards the healthy side, the particles move towards the cupula, which will undergo an ampullopetal (stimulatory) deflection, triggering a more intense apogeotropic nystagmus.

This BPPV variant may be caused by either cupulolithiasis, which manifests as apogeotropic persistent horizontal nystagmus [20], or, less frequently, by canalolithiasis, when the otoliths are located in the short arm of the horizontal semicircular canal, near the ampulla [18]. Cupulolithiasis (Figure 3(a)) is thought to play a greater role in horizontal canal BPPV than in the posterior canal variant and accounts for approximately 1/3 of the cases [14]. As otoconia is directly adherent to the cupula, the vertigo is intense and persists while the head is in the provocative position. When the patient's head is turned toward the affected side (Figure 3(b)), the cupula will undergo an ampullofugal (inhibitory) deflection causing an apogeotropic nystagmus. Turning the head to the opposite side (Figure 3(c)) will result in ampullopetal (stimulatory) deflection, manifesting as a stronger apogeotropic nystagmus. To summarize, horizontal canal BPPV owed to cupulolithiasis manifests as bilateral apogeotropic horizontal nystagmus, which is more pronounced on the healthy side. This is explained by Ewald's second law [21], according to which excitation of the horizontal canal is a more potent stimulus than inhibition. In several cases, instead of cupulolithiasis, canalolithiasis of the short arm of the horizontal semicircular canal near the ampulla may occur [18], presenting with similar nystagmus (bilateral apogeotropic) as in the cupulolithiasis cases (Figure 4).

## 4. Unilateral Anterior Canal BPPV

**Figure 5:**
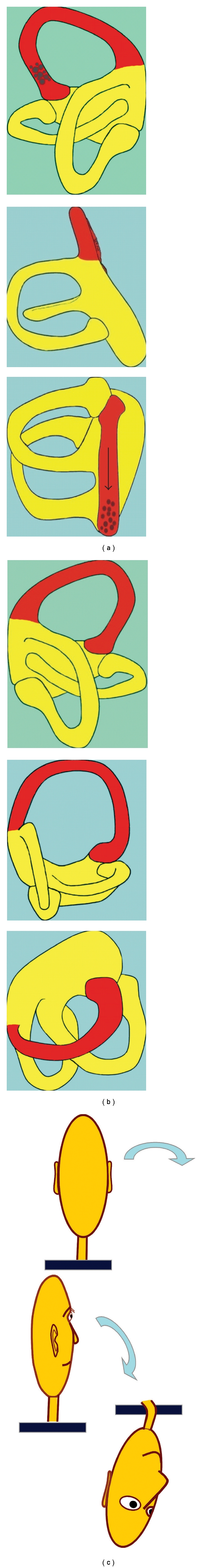
Stimulation of the left anterior semicircular canal during the right Dix-Hallpike maneuver (c). On (a,b), the semicircular canals of the left and right ears, respectively, are shown. When otoconia is present in the contralateral (left) anterior canal (a), the affected anterior canal is stimulated due to the movement of endolymph that takes place in its rotation plane, inducing BPPV.

**Figure 6:**
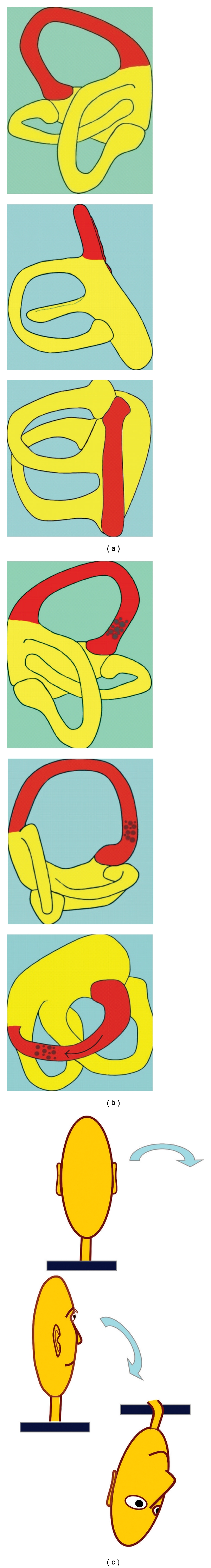
Stimulation of the right anterior semicircular canal during the right Dix-Hallpike maneuver (c). On (a,b), the semicircular canals of the left and right ears, respectively, are shown. When otoconia is present in the ipsilateral (right) anterior canal (b), the head rotates orthogonally to the plane of the affected anterior canal. However, due to the almost vertical orientation of the its ampullary segment, displacement of otoconia is induced as well and the test will be positive, although the provoked pressure against the cupula and the corresponding symptoms are expected to be less pronounced.

Anterior canal BPPV is quite rare and its incidence has been reported to range from 1-2% to 15% [6, 22]. It has been found that anterior canal BPPV produces bilaterally positive Dix-Hallpike maneuvers [23]. During a contralateral Dix-Hallpike maneuver (Figure 5), the head rotates in the plane of the affected anterior canal whereas during an ipsilesional Dix-Hallpike maneuver the head rotates orthogonally to the plane of the anterior canal (Figure 6). On both instances, the maneuver will be positive, due to the almost vertical orientation of the ampullary segment of the anterior canal. During the contralateral Dix-Hallpike test, the affected anterior canal is stimulated due to the movement of endolymph that takes place in its rotation plane. During the ipsilesional Dix-Hallpike test, the ampullary segment of the canal will also point downwards at about 40° off vertical. Consequently, displacement of otoconia in the involved anterior canal is induced and the test will be positive as well, although the provoked pressure against the cupula and the corresponding symptoms are expected to be less pronounced. 

In anterior canal BPPV, the observed nystagmus when the patient is moved into the Dix-Hallpike position is mixed, with the direction of the fast phase being downbeating and torsional [24]. When the direction of the torsional element of nystagmus is geotropic (superior pole of the eye moving toward the downside ear: counterclockwise on the right maneuver and clockwise on the left), excitation of the anterior canal of the downside ear may be inferred because debris in the ipsilateral affected anterior canal shifts to the most dependent position within the long arm of the canal, producing movement of the endolymph and excitation of the hair cells. This results in a torsional geotropic (right counterclockwise and left clockwise) and downbeating nystagmus. When the nystagmus is downbeating and torsional, but the fast phase of the torsional component is apogeotropic (superior pole of the eye beating toward the upside ear: clockwise on the right maneuver and counterclockwise on the left), it may be concluded that the affected anterior canal is in the upper ear. The differentiation between anterior and posterior BPPV should be based on the direction of the vertical component of the nystagmus. If the nystagmus is downbeating, the anterior canal of either ear may be affected, and conversely, if the nystagmus is upbeating, involvement of the posterior canal of the downside ear may be inferred. 

Additionally, it should be noticed that the torsional nystagmic component is smaller for the anterior than the posterior canal nystagmus, because the anterior canals are placed nearer to the sagittal plane, in comparison with the posterior canals [23]. Accordingly, there is an upwards bias in vertical slow phase eye velocity, and more downbeat than torsional nystagmus is expected from anterior canal BPPV and more torsional than upbeat nystagmus in posterior canal BPPV. It has been observed in several instances, that the torsional component may be completely absent in anterior canal BPPV, and the disease may manifest as pure downbeating nystagmus, mimicking a central nervous system disorder. In this case, localization of the side of the lesion based on the produced nystagmus is not possible. Finally, it should be mentioned that occasionally, downbeating nystagmus may be seen during CRPs, caused by inappropriate (centripetal) movement of otoconia, indicating ineffective CRP, needing a repeat [18]. 

## 5. Multiple-Canal BPPV

Multiple-canal BPPV includes either involvement of the same canal on both sides or simultaneous involvement of different canals on the same or on both sides. It should be noticed that traumatic origin is quite common in multiple-canal BPPV, as previously reported [1, 4, 25, 26]. We should particularly think of and search for multiple-canal BPPV versus single canal BPPV when the patient has suffered head trauma. The specific types of multiple-canal involvement are further discussed.

## 6. Bilateral Posterior Canal BPPV

Bilateral posterior canal involvement is presumed when Dix-Hallpike maneuver is positive on both sides. However, care should be taken to avoid the erroneous diagnosis of pseudobilateral posterior canal BPPV as true bilateral BPPV [27]. The entity of unilateral mimicking bilateral BPPV was first described by Steddin and Brandt. According to these authors, inappropriate head positioning during testing of the unaffected ear causes displacement of the affected posterior canal from its perpendicular position. This makes the otolith debris move gravitationally towards the cupula, causing thus transient cupulolithiasis and evoking an inhibitory nystagmus. This nystagmus is directed towards the lower unaffected ear and this situation may be erroneously diagnosed as bilateral posterior canal BPPV. The inhibitory nystagmus usually has a lower amplitude and frequency than the excitatory nystagmus of the affected ear, and patients report less symptoms when the unaffected ear is tested. Additionally, the nystagmus during testing the noninvolved side may have a downbeating component and a longer duration [28].

Differential diagnosis between true bilateral and pseudobilateral posterior canal BPPV may be obtained based on the following [28, 29].

- The presence of asymmetric nystagmus and symptoms of different intensity between right and left Dix-Hallpike maneuvers should arouse the suspicion of pseudobilateral posterior BPPV. The side with more intense nystagmus and symptoms may probably be the affected side.
- Performance of a head-down test, extending the head of the patient directly backward from the sitting to the supine straight head hanging position, might be helpful. During this test, both posterior canals get irritated, resulting in the appearance of nystagmus. This nystagmus has only a vertical upbeating component, because the torsional components, having opposite directions, are cancelled. True bilateral BPPV may be concluded in this case, whereas in case that the nystagmus retains its torsional component, pseudobilateral BPPV is probable. The true side of the disease may be found, observing the direction of this component, which beats clockwise on left posterior canal BPPV and Counterclockwise on right posterior canal involvement.
- The criterion of responsiveness to treatment is quite helpful. Successful treatment of the patient after performing the appropriate CRP on the side with more intense manifestations is proof of previously pseudobilateral BPPV [27]. When it is necessary to repeat the CRP contralaterally to obtain remission of the symptoms, this may be proof of bilateral posterior BPPV.

## 7. Bilateral Horizontal Canal BPPV

Bilateral horizontal canal BPPV is quite difficult to diagnose. The critical point in this case is that during the supine roll test, unilateral horizontal canal BPPV elicits horizontal nystagmus on both sides, either geotropic (canalolithiasis mechanism) or apogeotropic (cupulolithiasis and canalolithiasis mechanism) [2].

### 7.1. Geotropic Bilateral Horizontal Canal BPPV

**Figure 7:**
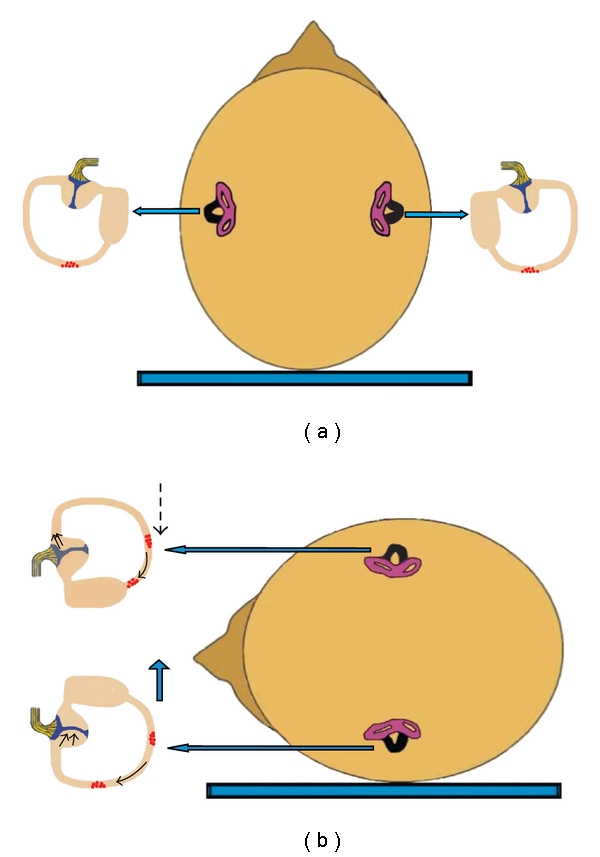
Mechanism of canalolithiasis of bilateral geotropic horizontal canal BPPV (the involved horizontal canal sare colored black). (a) Patient in supine position with debris in the posterior part of both horizontal canals. (b) Supine roll test on either side would result in excitation of the horizontal canal of the lowermost ear, due to ampullopetal endolymph flow and at the same time inhibition of the horizontal canal of the uppermost ear, due to ampullofugal endolymph flow. Vectorial summation would result in an intense, symmetric geotropic nystagmus.

In a theoretical case of bilateral horizontal BPPV with geotropic nystagmus, supine roll test on either side would result in excitation of the horizontal canal of the lowermost ear, due to ampullopetal endolymph flow and at the same time inhibition of the horizontal canal of the uppermost ear, due to ampullofugal endolymph flow (Figure 7). Vectorial summation would result in an intense, symmetric geotropic nystagmus.

### 7.2. Apogeotropic Bilateral Horizontal Canal BPPV

**Figure 8:**
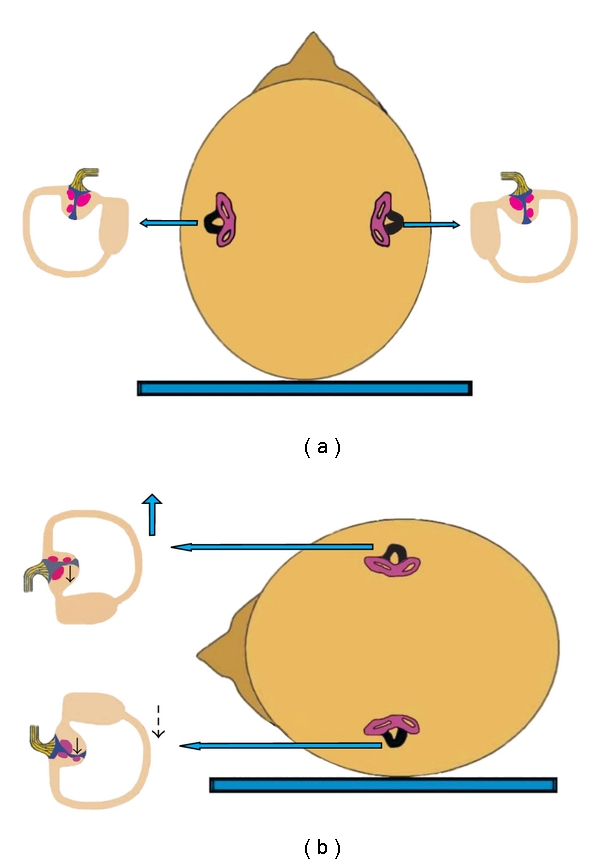
Mechanism of cupulolithiasis of bilateral apogeotropic horizontal canal BPPV (the involved horizontal canals are colored black). (a) Patient in supine position with debris adherent to the cupula of both horizontal canals. (b) Supine roll test on either side would result in inhibition of the horizontal canal of the lowermost ear, due to ampullofugal (inhibitory) deflection, triggering an apogeotropic horizontal nystagmus. At the same time, excitation of the horizontal canal of the uppermost ear would occur, due to ampullopetal (stimulatory) deflection of the cupula. Vectorial summation would result in an intense, more or less symmetric, apogeotropic nystagmus.

In a hypothetical case of bilateral horizontal BPPV with apogeotropic nystagmus, supine roll test on either side would result in inhibition of the horizontal canal of the lowermost ear, due to ampullofugal cupular movement, triggering an apogeotropic horizontal nystagmus. At the same time, excitation of the horizontal canal of the uppermost ear, due to ampullopetal movement of the cupula, would occur (Figure 8). Vectorial summation would result in an intense, more or less symmetric, apogeotropic nystagmus. It may be assumed that the nystagmus would be more intense in apogeotropic bilateral horizontal canal BPPV because of a dual pathogenetic mechanism: inhibition of the horizontal canal of the lower ear and, concurrently, excitation of the horizontal canal of the upper ear. In comparison, in cases with unilateral involvement of the horizontal canal, only one mechanism contributes to the produced apogeotropic nystagmus: either inhibition of the affected ear on turning towards its direction or excitation of the affected ear on turning towards the healthy ear. The same mechanism is valid in cases with canalolithiasis of the ampullary arm of the horizontal canal.

**Figure 9:**
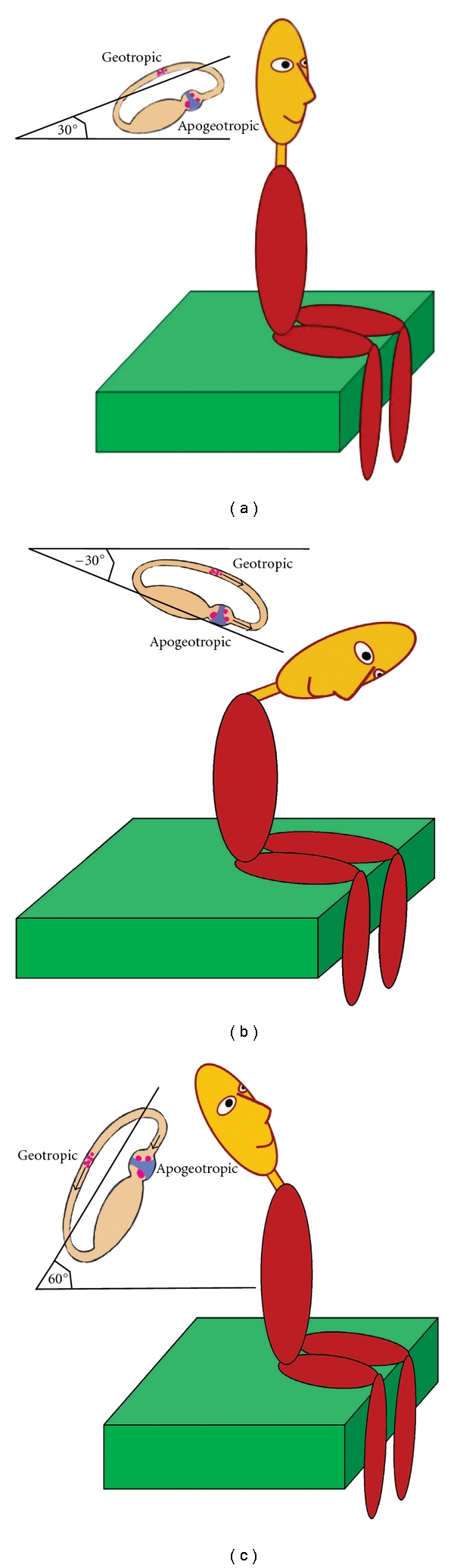
Pseudospontaneous nystagmus in horizontal canal BPPV. (a) When the head is erect, there is an angle of 30° between the horizontal plane and the horizontal semicircular canal, which may explain the pathogenesis of pseudospontaneous nystagmus. Any head movements would cause ampullofugal movement of otoconia, in case of canalolithiasis (geotropic type), provoking a nystagmus with fast phase towards the unaffected ear. In case of cupulolithiasis (apogeotropic type), the attached otoconial mass would cause deflection of the cupula in the opposite direction, triggering a reverse nystagmus. (b) When the head is bent 60° forward, pseudospontaneous nystagmus may change its direction, due to the inversion of the movement of otoconia (geotropic type) or of the deflection of the cupula (apogeotropic type). (c) When the head is bent backwards, pseudospontaneous nystagmus will increase, because the horizontal canal will approach the vertical position.

It has been reported that 10% of the cases with unilateral horizontal BPPV may present with symmetrical nystagmus [30]. In this case it is difficult to detect the side of the lesion. To accomplish this, study of the pseudospontaneous nystagmus with the head pitch test has been proposed [31]. It has been reported that patients with horizontal BPPV may exhibit a spontaneous horizontal nystagmus while in the sitting position. This represents probably a pseudospontaneous nystagmus because it is strongly influenced by head position and movements. When spontaneous nystagmus is absent, it is occasionally possible to evoke it with mild horizontal movements of the head. Pathogenesis of the pseudospontaneous nystagmus may be explained by the angle of 30° which exists between the horizontal plane and the horizontal semicircular canal, when the head is erect. Any head movements, even if minimal, would cause free debris floating inside the canal to move away from the ampulla, provoking a nystagmus with fast phase towards the unaffected ear. In case of cupulolithiasis, the attached otoconial mass would cause movement of the cupula in the opposite direction, triggering a reverse nystagmus (Figure 9(a)).

By bending the head 30° forward, spontaneous nystagmus should disappear because the horizontal canal assumes a true horizontal position, and either free debris or the heavy cupula is not further influenced by the gravity vector. Furthermore, by bending the headforward to about 60° (Figure 9(b)), gravity causes ampullopetal movement of the debris, resulting in a nystagmus fast phase towards the affected ear, which is the opposite direction from that observed with the head erect. If the otoconia is attached to the cupula, the cupular deflection will be in the opposite direction, triggering an opposite nystagmus (towards the unaffected ear). Finally, backward bending of the head (Figure 9(c)) will cause an increase in spontaneous nystagmus, because the canal will be approximately in the vertical position, similar to the position at which we locate the patient to perform calorics. This type of testing is called “bow and lean test” [21], or more appropriately head pitch test [31], and has been used to determine the affected side, once we know from the previous maneuvers that the positional horizontal nystagmus is of the geotropic or the apogeotropic type. 

**Figure 10:**
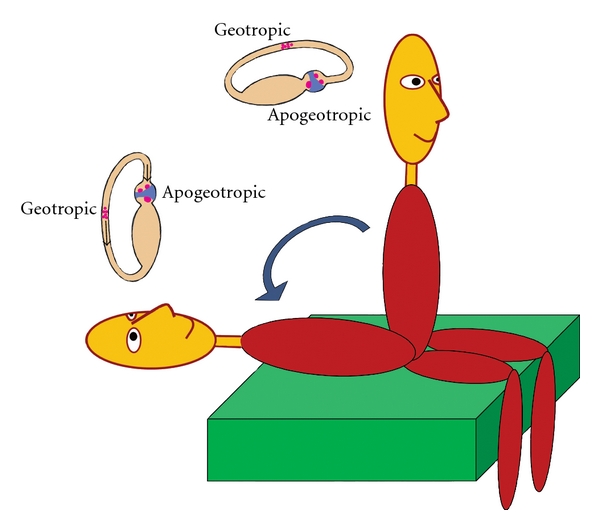
When the patient with horizontal canal BPPV is quickly brought from the sitting to the supine position, a mild horizontal nystagmus may appear, attributed either to ampullofugal movement of otoconia in the horizontal canal (geotropic type), triggering nystagmus toward the unaffected ear, or in cases of cupulolithiasis (apogeotropic type) to ampullopetal deflection of the cupula, resulting in nystagmus directed toward the affected ear.

Another method is to examine the appearance of positional nystagmus by performing the head down test [32, 33], quickly bringing the patient from the sitting to the supine position, in the sagittal plane (Figure 10). Frequently, a mild horizontal nystagmus appears, attributed to the movement of debris in the horizontal canal, when canalolithiasis is the underlying pathology. This movement causes the debris to move ampullofugally, resulting in nystagmus towards the unaffected ear. In cases of cupulolithiasis, otoconial debris attached to the cupula causes ampullopetal movement, resulting in nystagmus directed towards the affected ear. 

In cases of bilateral symmetrical nystagmus, owed to bilateral involvement, neither pseudospontaneous nystagmus nor nystagmus during the head down test should be present most of the time. However, if some type of nystagmus could be observed, its direction should not be stable, but changing according to the prevailing movement of otoconia in each horizontal canal. It should be further noticed that the criterion of symmetry of the nystagmus to diagnose bilateral horizontal canal BPPV is not a solid one, because asymmetric involvement of the two horizontal canals may occur as well. Combination of canalolithiasis-cupulolithiasis, either on the same or on different horizontal canals, would complicate the matter further. It has been reported though, that reversal of the geotropic nystagmus to apogeotropic, while the subject remains in the same lateral position during the supine roll test, may be explained from concomitant canalolithiasis-cupulolithiasis in the horizontal canal of the lower ear [19].

It may be thus concluded that although patients with bilateral disease of the horizontal canal may exist, difficulty in diagnosis may explain why cases with this type of vertigo have been scarcely reported. Horii et al. [34] described such an interesting case of bilateral horizontal BPPV, treated successfully by canal plugging of the horizontal canal on one side and the Lempert maneuver on the other side.

## 8. Bilateral Anterior Canal BPPV

Bilateral anterior canal is also very difficult to diagnose. Theoretically, the Dix-Hallpike maneuver on the right side would cause paroxysmal nystagmus with a vertical downbeating component and a torsional component with the upper pole of the eye beating clockwise (at opposite direction of the posterior canal BPPV). This type of nystagmus is attributed to excitation of the contralateral anterior canal. However, as previously discussed, the ipsilateral anterior canal would be also excitated, resulting in a downbeating vertical component, and a torsional in the opposite direction, but probably of a smaller intensity [35]. Vectorial summation of all the components would result in an intense vertical downbeating component and a weak torsional component towards the upper ear. In conclusion, the Dix-Hallpike maneuvers on both sides would produce a mixed nystagmus, with an intense vertical and a weak torsional component on both occasions opposite to those of posterior canal involvement. Differential diagnosis would be difficult because in unilateral anterior BPPV, the torsional nystagmic vector may be quite often absent. 

Furthermore, in a head down test, performed in a similar way as in the case of bilateral posterior canal BPPV, both anterior canals would get irritated again, resulting in the appearance of nystagmus. This nystagmus should be characterized by only an intense purely vertical downbeating component, because the torsional components would cancel each other, as having opposite directions. Differential diagnosis on these grounds would not be safe, because unilateral anterior canal involvement, as previously mentioned, may manifest as purely vertical as well. Finally, the criterion of responsiveness to treatment is not helpful in this case. There is not established therapeutic maneuver for treatment of the anterior canal BPPV, and both standard Epley and reverse Epley maneuvers have been used, as well as various specifically designed maneuvers [36, 37]. To conclude, although the entity of bilateral anterior BPPV has been previously reported [4, 22], any details concerning its diagnosis and treatment are missing. We believe that this diagnosis is presently highly hypothetical. 

## 9. Horizontal and Posterior Canal BPPV

This is the most common case of mixed canal BPPV [37–39]. The involved canals may be either on the same side or on both sides. Diagnosis may be easily obtained, considering the features of the nystagmus on either maneuver. Mixed torsional geotropic-vertical upbeating nystagmus in Dix-Hallpike maneuvers reveals involvement of the posterior canal. Additionally, geotropic or apogeotropic horizontal nystagmus during the supine roll test will be evidence of horizontal canal BPPV. In most mixed cases, the horizontal nystagmus is geotropic due to canalolithiasis, although cupulolithiasis has been occasionally reported [40]. It should be noticed, however, that during the Dix-Hallpike tests, the horizontal canal is also, at least, partially stimulated, and a horizontal component of nystagmus may be evident in conjunction with the torsional-upbeating nystagmus of posterior canal origin [41]. Additionally, it has been shown that horizontal nystagmus, provoked during the supine roll test, exhibits also a vertical and a torsional component [42].

## 10. Horizontal and Anterior Canal BPPV

Occurrence of this combination is quite unusual, due to rare involvement of the anterior canal. Diagnosis of horizontal canal involvement is evident, according to previously described characteristics of the nystagmus. Anterior canal BPPV may be also diagnosed from the experienced clinician, as already described. The main feature for differential diagnosis from the posterior canal BPPV is the downbeating vertical component of the nystagmus. Additionally, the direction of the torsional component will show if either the ipsilateral or the contralateral canal is involved. 

## 11. Posterior and Anterior Canal BPPV

This combination has also been reported [4, 22], but its diagnosis presents difficulties. Two categories of involvement should be distinguished, on the same side and on different sides.

- If the involved posterior and anterior canals are on the same side, then Dix-Hallpike on this side would cause theoretically (1) a torsional component with the upper eye pole moving geotropically and a vertical upbeating component, due to posterior canal disease; (2) a torsional component with the same direction and a vertical downbeating component, due to anterior canal disease. The net result would be only a strong torsional component, because the two torsional components would be added and the two vertical components would be cancelled, as having opposite directions. Dix-Hallpike on the nonaffected ear would cause a torsional component with the upper eye pole moving apogeotropically and a vertical downbeating component, due to anterior canal disease, as previously discussed.
- If the involved posterior and anterior canals are on different sides, then Dix-Hallpike on the side where the posterior canal is involved, theoretically would cause (1) a torsional component with the upper eye pole moving geotropically and a vertical upbeating component, due to posterior canal disease; (2) a torsional component with opposite direction and a vertical downbeating component, due to anterior canal disease of the contralateral side. The net result will be absence of nystagmus, due to vectorial subtraction of the partial components. However, some asymmetry of canal involvement may manifest as mild nystagmus, either torsional, or vertical, or mixed, depending on the intensity of the partial components. Dix-Hallpike on the side of the involved anterior canal would cause a mixed nystagmus, with a torsional component with the upper eye pole moving geotropically and a vertical downbeating component, due to ipsilateral anterior canal disease.

From what has been mentioned above, it is understandable why mixed posterior-anterior canal is so difficult to be diagnosed with certainty. In case of suspicion, separate CRPs for the posterior canal (mainly the Epley CRP) and a specific therapeutic procedure for the anterior canal [35] could support the diagnosis, if treatment could be obtained. However, it should be noticed that an Epley maneuver for posterior canal BPPV is also a reverse Epley (and probably therapeutical) for the contralateral anterior canal, thus complicating this issue further.

## 12. Conclusions

- Typical posterior canal BPPV and horizontal canal BPPV are usually easy to diagnose, using the standard Dix-Hallpike and supine roll maneuvers, respectively.
- Anterior canal BPPV presents difficulties in diagnosis because it may demonstrate mixed vertical-torsional nystagmus on both right and left Dix-Hallpike maneuvers. Additionally, the torsional nystagmic component may be missing.
- BPPV involving both posterior canals may be easily detected, but it is almost impossible to diagnose in cases of bilateral horizontal or anterior canal involvement.
- BPPV involving two different canals, either on the same or on different sides, may be quite safely diagnosed in typical cases of posterior-horizontal and anterior-horizontal involvement. However, the combination of posterior-anterior canal involvement is more difficult to diagnose with certainty.

## Figures and Tables

**Table 1a:** (a) Vertical SC canals

Involved SC canal	Diagnostic maneuver	Paroxysmal positioning nystagmus	
		Vertical	Torsional
P-BPPV R	Dix-Hallpike R (+)	Upbeating	Counterclockwise
	Dix-Hallpike L (−)	No nystagmus	

P-BPPV L	Dix-Hallpike R (−)	No nystagmus	
	Dix-Hallpike L (+)	Upbeating	Clockwise

A-BPPV R	Dix-Hallpike R (+)	Downbeating	Counterclockwise
	Dix-Hallpike L (+)	Downbeating	Counterclockwise

A-BPPV L	Dix-Hallpike R (+)	Downbeating	clockwise
	Dix-Hallpike L (+)	Downbeating	Clockwise

**Table 1b:** (b) Horizontal SC canals

		Direction of nystagmus	Intensity of nystagmus	Pathogenetic mechanism
H-BPPV R	Supine roll test R (+)	Geotropic	More intense	Canalolithiasis
	Supine roll test L (+)	Geotropic	Less intense	

H-BPPV R	Supine roll test R (+)	Apogeotropic	Less intense	Cupulolithiasis or canalolithiasis of the short arm of the horizontal SC
	Supine roll test L (+)	Apogeotropic	More intense	

H-BPPV L	Supine roll test R (+)	Geotropic	Less intense	Canalolithiasis
	Supine roll test L (+)	Geotropic	More intense	

H-BPPV L	Supine roll test R (+)	Apogeotropic	More intense	Cupulolithiasis or canalolithiasis of the short arm of the horizontal
	Supine roll test L (+)	Apogeotropic	Less intense	
